# Distal end of a ureteral double-J stent displaced into the contralateral ureter after percutaneous nephrolithotripsy: a case report

**DOI:** 10.3389/fmed.2023.1239053

**Published:** 2023-09-21

**Authors:** Junyong Chen, Tao Xie, Ya Xu, Jian Deng, Jie Zhang, Yumo Zhu, Guiyuan Li

**Affiliations:** ^1^Department of Urology, Zhuhai People's Hospital (Zhuhai Hospital Affiliated with Jinan University), Zhuhai, China; ^2^Department of Radiology, Zhuhai People's Hospital (Zhuhai Hospital Affiliated with Jinan University), Zhuhai, China

**Keywords:** distal end, ureteral double-J stent, displacement, contralateral ureter, PCNL, case report

## Abstract

We report displacement of the distal end of a ureteral double-J stent into the contralateral ureter after percutaneous nephrolithotripsy (PCNL) in a 71-year-old man with a history of left kidney stones. Postoperative computed tomography imaging showed that the distal end of the left ureteral double-J stent was displaced into the right ureter, which resulted in persistent right renal colic when the nephrostomy tube was clipped and continuous urine leakage from the nephrostomy opening after the nephrostomy tube was removed. After the cystoscopic adjustment of the ureteral stent, the patient recovered uneventfully and was discharged home the next day.

## Introduction

A ureteral stent placement is a standard procedure after percutaneous nephrolithotripsy (PCNL) to maintain urine drainage, prevent post-surgery stenosis, and promote ureteral recovery from injuries (1). In most cases, ureteral stents are placed under the guidance of an endoscope, with or without the confirmation of intraoperative radiology. It has been reported that the distal and proximal migration of ureteral stents is not rare in clinical practice (2), which usually needs no surgical interventions. Moreover, there were cases reporting the displacement of ureteral stents in the inferior vena cava (3), ventricle (4), atrium (5), duodenum (6), rectum (7), peritoneum (8), etc., and close monitoring was warranted. However, the migration of a double-J stent in the contralateral ureter has rarely been reported before, which is also hard to distinguish and may cause non-specific symptoms including lumbar pain and hydronephrosis (9). In this study, we report a rare case in which the distal end of the ureteral stent was displaced into the contralateral ureter, which has never been reported in PCNL anywhere before to our knowledge. We believe that this case report could improve our awareness of the possible complications related to PCNL and better guide clinical practice in similar situations.

## Case description

**Figure 1:**
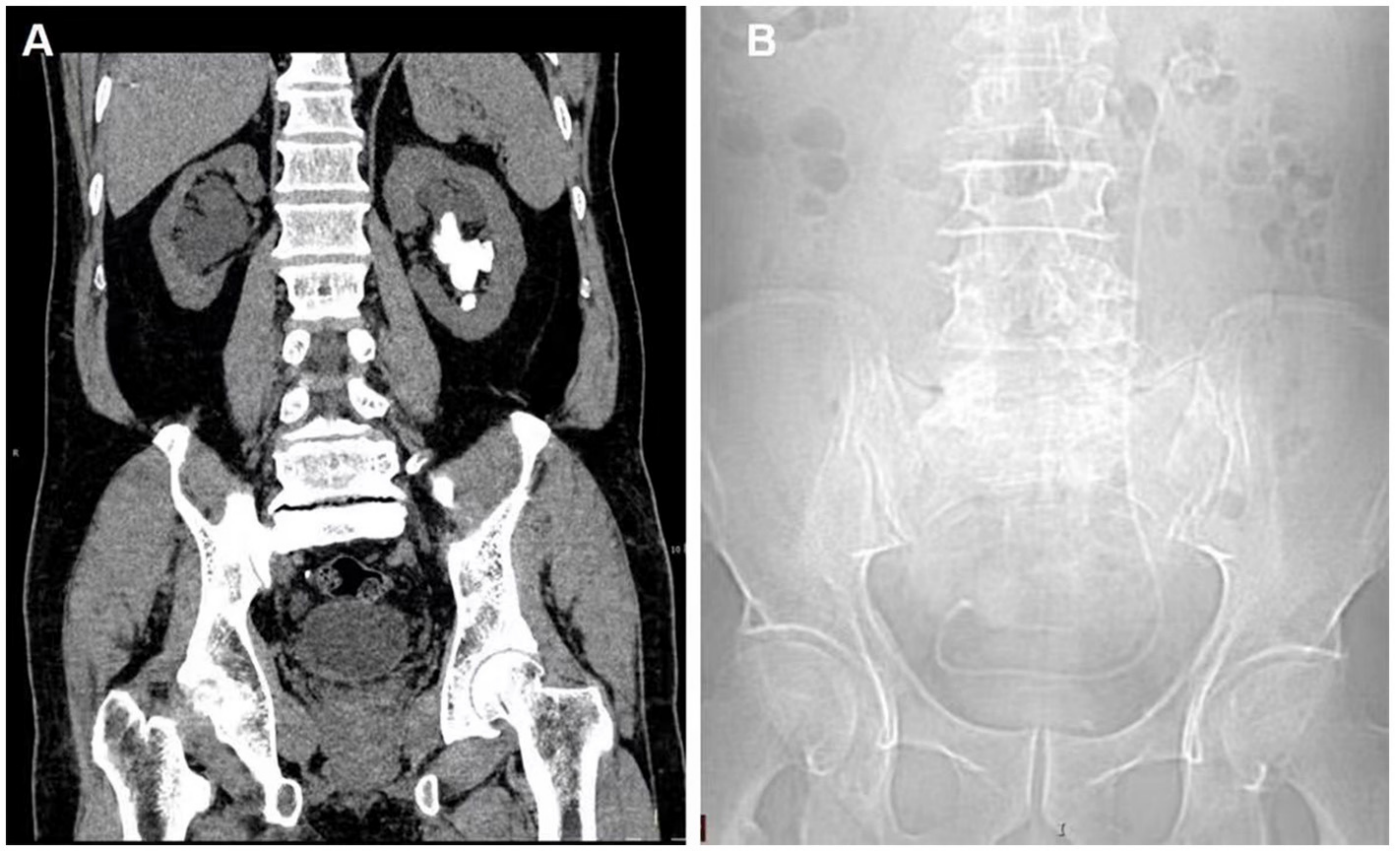
**(A)** CT examination before PCNL indicated left kidney stones and hydronephrosis of the right kidney. **(B)** Plain X-ray after PCNL showed the location of the left ureteral stent.

**Figure 2:**
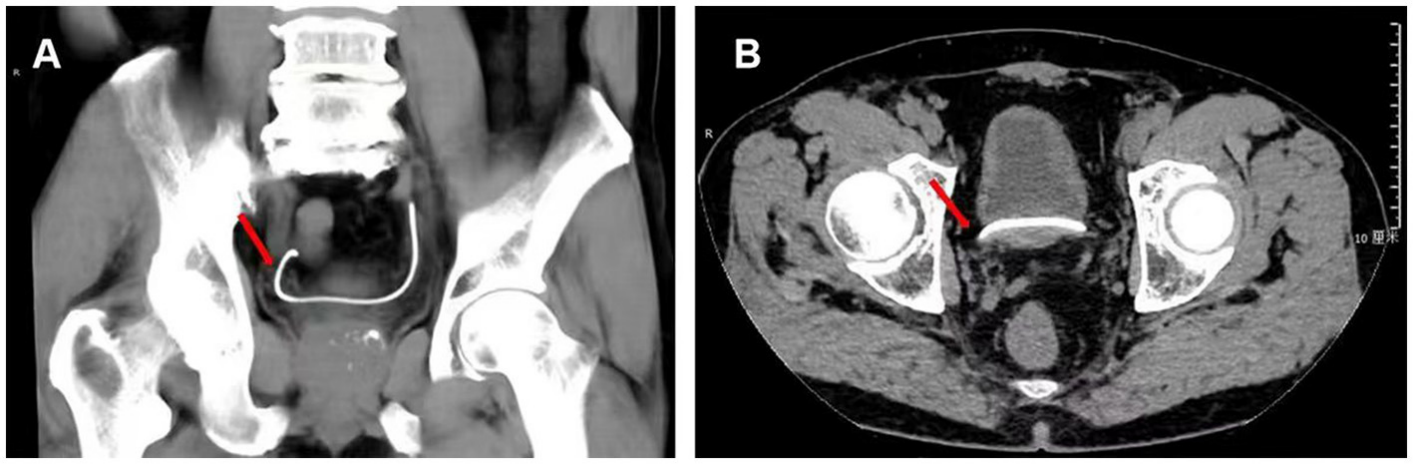
CT examination after PCNL revealed that the distal end (red arrow) of the left ureteral stent was displaced beyond the bladder and into the right ureter. **(A)** Coronary section. **(B)** Axial section.

On 6 March 2021, a 71-year-old man was admitted to our hospital for urinary frequency and hematuria with a history of kidney stones. Computed tomography (CT) indicated that the patient had a left kidney stone (Figure 1A). The patient subsequently underwent an uneventful left PCNL and had a left ureteral stent inserted. He was sent back to the ward with his nephrostomy tube clipped for the purpose of hemostasis, and soon experienced persistent right renal colic even after conservative intervention, although preoperative CT showed no calculi in his right urinary system. On day 1 after the surgery, the patient's colic was miraculously relieved when the nephrostomy tube was opened. On day 2, the patient received a plain X-ray examination, and the left ureteral stent was thought to be in a “normal” position (Figure 1B): The proximal end of the stent was in the kidney area, and the distal end was in the “bladder area.” Thus, we decided that the patient might suffer from transient right renal colic as a result of vesicoureteral reflux, which was related to an overactive bladder and a dilation of the right ureter as indicated by preoperative CT. On day 3, the patient complained of continuous urine leakage from the nephrostomy opening shortly after the removal of the nephrostomy tube. On day 4, the patient received a CT examination, which showed that the distal end of the left ureteral stent was displaced into the right ureter (Figures 2A, B).

**Figure 3:**
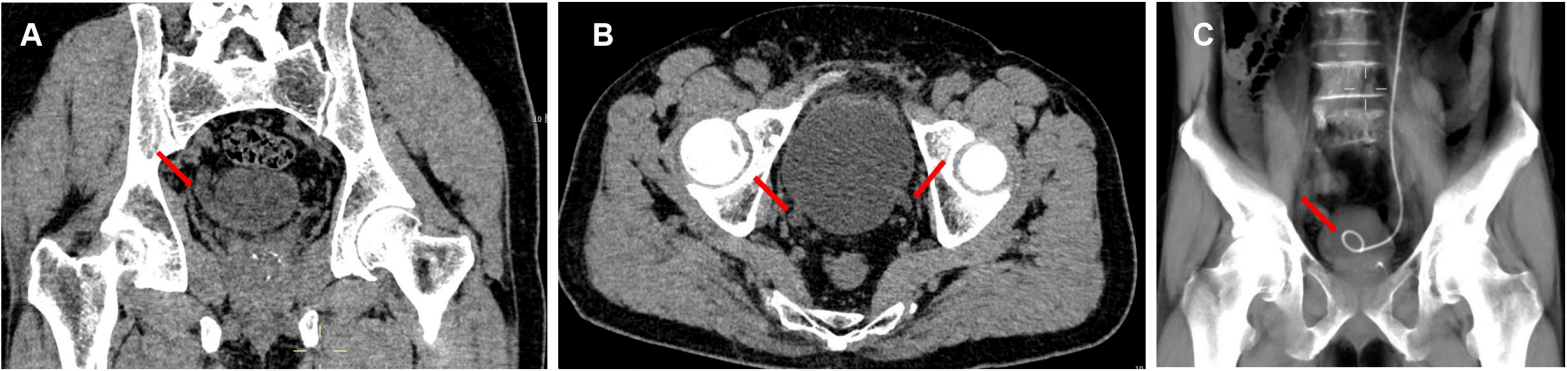
**(A)** Coronary section of CT examination before PCNL indicated the dilation of right ureter (red arrow). **(B)** Axial section of CT examination before PCNL showed the dilation of bilateral ureters (red arrows). **(C)** CT examination showed the location of left ureteral stent after the adjustment guided by cystoscope.

After discussion, we agreed that urgent intervention should be initiated for the adjustment of the ureteral stent. On day 5, the decision was made to proceed with cystoscopy, and the distal curl was not observed within the bladder but lying into the contralateral ureter. The double-J stent was removed, and a new one was placed back into the left ureter, with the location of the stent being confirmed by postoperative CT (Figure 3C). The patient was discharged home 6 h after the cystoscopy, with his right renal colic and urine leakage relieved. Subsequent follow-up revealed that the patient had an uneventful recovery with good tolerability, and came back to our hospital for day surgery to remove the double-J stent 4 weeks later.

## Discussion

Ureteral stents have been used as a temporary or permanent solution for ureteral obstruction (10). The ureteral stent placement can be antegrade or retrograde. In this case, a left ureteral stent is placed using an antegrade approach after PCNL.

Ureteral stent placement might cause various complications, including encrustation, fracture, and proximal and distal displacement within the ipsilateral ureter (11). Under most circumstances, the displacement within the urinary system was asymptomatic and required no urgent intervention. The migration of a double-J stent in the contralateral ureter was extremely rare. We found only one similar case report in which the double-J stent migrated in the contralateral ureter during robot-assisted pyeloplasty, which caused hydronephrosis and lumbar pain (9).

In our case, the distal end of the ureteral stent was displaced into the contralateral ureter through a guidewire, which was placed excessively in an antegrade way and incidentally inserted into the contralateral ureteral orifice. As a consequence, the patient suffered from right renal colic and urine leakage from the left nephrostomy opening due to the occlusion of the ureter lumen by the double-J stent. So far as we are concerned, this rare migration of the stent has never been described in PCNL. After early surgical intervention, no severe damages have been made, and the patient expressed his understanding of the migration of the stent and approved the medical team's timely treatment.

We proposed that several factors may result in the displacement of the ureteral stent: (1) short ureter and closely located ureteral meatus: the patient was only 160 cm tall, which implied a shorter ureter, preoperative CT indicated closely located and significantly dilated orifices of bilateral ureters (Figures 3A, B); (2) excessive placement of guidewire: zebra guidewire was placed excessively in an antegrade way and incidentally inserted into the contralateral ureter, thus leading to the displacement of the double-J stent; (3) delayed confirmation of double-J stent: plain X-ray was performed on day 2 after the surgery, although right renal colic was not relieved after the conservative intervention, and (4) insufficient awareness of the rare migration of double-J stent: due to the rarity of the relevant case report, we failed to identify the migration in the first place, and neglected the truth hidden behind the refractory right renal colic.

Admittedly, there are certain limitations to our report. The data on drainage volume for the nephrostomy tube and urinary catheter were not available. The intraoperative photos featuring the ureteral orifices and the displacement of the ureteral stent were also missing. However, the main concern of the displacement was not the transient damage to the patient's renal function or symptoms but delayed recovery of the percutaneous renal passage, potential infection, and low quality of life due to continuous urine leakage. Therefore, we believed that this case report was valuable in clinical settings.

## Conclusion

We first report a case in which the distal end of a double-J stent migrated in the contralateral ureter after percutaneous nephrolithotripsy, resulting in postoperative renal colic and continuous urine leakage from the nephrostomy opening. This rare migration possibly results from a short ureter, closely located bilateral ureteral orifices, and excessive placement of the guidewire in an antegrade way. For this rare complication, surgical intervention should be initiated as soon as possible. Prevention strategies include proper placement of the guidewire, timely confirmation by radiology after the placement of the ureteral stent, careful evaluation of postoperative symptoms, and early intervention.

## Data availability statement

The original contributions presented in the study are included in the article/supplementary material, further inquiries can be directed to the corresponding author.

## Ethics statement

Written informed consent was obtained from the individual(s) for the publication of any potentially identifiable images or data included in this article.

## Author contributions

JC and YX designed the manuscript. TX, JZ, and YZ collected the clinical data and provided the materials. JD revised the manuscript. GL wrote the first draft of the manuscript. All authors participated in the diagnosis and treatment of the patient and read and approved the final manuscript.
